# Endoplasmic Reticulum Stress: An Emerging Therapeutic Target for Intervertebral Disc Degeneration

**DOI:** 10.3389/fcell.2021.819139

**Published:** 2022-02-01

**Authors:** Dong Wang, Xin He, Chao Zheng, Chengzhe Wang, Pandi Peng, Chu Gao, Xiaolong Xu, Yachao Ma, Mei Liu, Liu Yang, Zhuojing Luo

**Affiliations:** ^1^ Institute of Orthopedic Surgery, Xijing Hospital, Fourth Military Medical University, Xi’an, China; ^2^ Pharmacy Department, Air Force Hospital of Eastern Theater Command, Nanjing, China; ^3^ Rehabilitation Department, Dongchangfu Traditional Chinese Medicine Hospital, Liaocheng, China; ^4^ Institute of Flexible Electronics, Northwestern Polytechnical University, Xi’an, China; ^5^ Medical Research Institute, Northwestern Polytechnical University, Xi’an, China

**Keywords:** low back pain, intervertebral disc degeneration, endoplasmic reticulum, unfolded protein response, cellular homeostasis

## Abstract

Low back pain (LBP) is a global health issue. Intervertebral disc degeneration (IDD) is a major cause of LBP. Although the explicit mechanisms underpinning IDD are unclear, endoplasmic reticulum (ER) stress caused by aberrant unfolded or misfolded proteins may be involved. The accumulation of unfolded/misfolded proteins may result in reduced protein synthesis and promote aberrant protein degradation to recover ER function, a response termed the unfolded protein response. A growing body of literature has demonstrated the potential relationships between ER stress and the pathogenesis of IDD, indicating some promising therapeutic targets. In this review, we summarize the current knowledge regarding the impact of ER stress on the process of IDD, as well as some potential therapeutic strategies for alleviating disc degeneration by targeting different pathways to inhibit ER stress. This review will facilitate understanding the pathogenesis and progress of IDD and highlights potential therapeutic targets for treating this condition.

## Introduction

According to a systematic analysis by the Global Burden of disease study 2019, approximately 80% of individuals experience low back pain (LBP) during their lifetime. For the increased population and aging, disability-adjusted life-years (DALYs) caused by LBP increased by 46.9% between 1990 and 2019. Because of the highest prevalence and years of life lived with disability (YLDs) of all musculoskeletal diseases, LBP has caused a substantial socioeconomic burden on society (Diseases and Injuries, 2020; Cieza et al., 2021). Intervertebral disc degeneration (IDD), which is characterized by abnormalities in local physiological structure [reduced hydration of the nucleus pulposus (NP), disorganization of the annulus fibrosus (AF), and calcification of the cartilage endplates (CEP)] and radiological evidence [decreased disc height in radiography and reduced T2 signal intensity in magnetic resonance imaging (MRI)], is a major cause of LBP (Luoma et al., 2000; Brinjikji et al., 2015; Von Forell et al., 2015; Zheng and Chen, 2015; Luoma et al., 2016; van den Berg et al., 2017; Taylor and Bishop, 2019). The commonly used clinical therapeutic methods focus on symptomatic relief from pain by conservative treatments (restricted exercise, low-tension traction, physical therapy, injections, etc.) or surgical measures (disc decompression, disc replacement or spinal fusion) (Raj, 2008; Urits et al., 2019). However, these interventions possess a number of limits. As final selections, surgical operations usually bring heavy cost, surgery risks and a long journey of rehabilitation to patients. While conservative treatment showed no effect on inhibiting the progression of IDD or restoring the dampened IVD structure (Raj, 2008; Urits et al., 2019; O'Keeffe et al., 2020; Meroni et al., 2021). Thus, seeking new therapeutic strategies for inhibiting IDD process is a burning question for clinical researchers.

Intervertebral discs (IVDs) consist of three basic anatomical parts: the inner NP, outer AF, and superior and inferior CEP, which connect the vertebrae and offer flexibility to the spine (Adams and Dolan, 2012; Gopal et al., 2012; Pattappa et al., 2012; Kepler et al., 2013; Vergroesen et al., 2015). The intact IVD elements help the spine resist mechanical stress and maintain the range of motion. While a dampened IVD structure disrupts the stability of spine and thus leads to the pathogenic progress of IDD (Urban et al., 2004; Inoue and Espinoza Orias, 2011). The pathogenesis of IDD is complex and includes various risk factors, such as genetic variation, disorganized immune system status, excessive loading, circadian rhythm disorders, and aging (Ma et al., 2015; Bian et al., 2017; Dudek et al., 2017; Kitis et al., 2018; Xu et al., 2019). At cellular and molecular levels, these risk factors induce abnormal mitochondrial and endoplasmic reticulum (ER) function, impair redox and immunity homeostasis, and ultimately trigger IVD cell senescence and apoptosis, resulting in IDD (Chen et al., 2016; Cheng et al., 2018; Luo et al., 2019a; Liao et al., 2019). Although numerous factors contribute to the process of IDD, our understanding of its molecular mechanisms remains limited.

A stable extracellular matrix (ECM) is critical for maintaining IVD homeostasis. In NP tissue, synthesized ECM, mainly proteoglycans and type-II collagens, is highly hydrated, which helps the IVDs resist the axial mechanical loading. While in AF tissue, type-I collagens constitute the main components of the ECM, which shows the ability to bear the lateral deformation (Vergroesen et al., 2015; Bian et al., 2017). At the subcellular level, the ER plays critical roles in the synthesis of ECM on account of its function for protein folding, lipid/ion transfer, signaling, and metabolism. However, various stimulus for IVDs, such as hypoxia, starvation, inflammation, abnormal mechanical loading, imbalances in reactive oxygen species (ROS), and perturbation of Ca^2+^ levels, may trigger ER stress by interrupting protein folding and/or modification and thus dampen the synthesis of ECM. The evidence mentioned above implies that ER stress may be involved in the process of IDD (Wu et al., 2018; Reibe and Febbraio, 2019; Carlton et al., 2020).

Here, we review the molecular basis for the sensing and response of ER stress, the involvement of ER stress in the pathogenesis of IDD, and some promising therapeutic strategies by targeting ER stress pathway.

## Molecular Basis for the Sensing and Response of ER Stress

**FIGURE 1:**
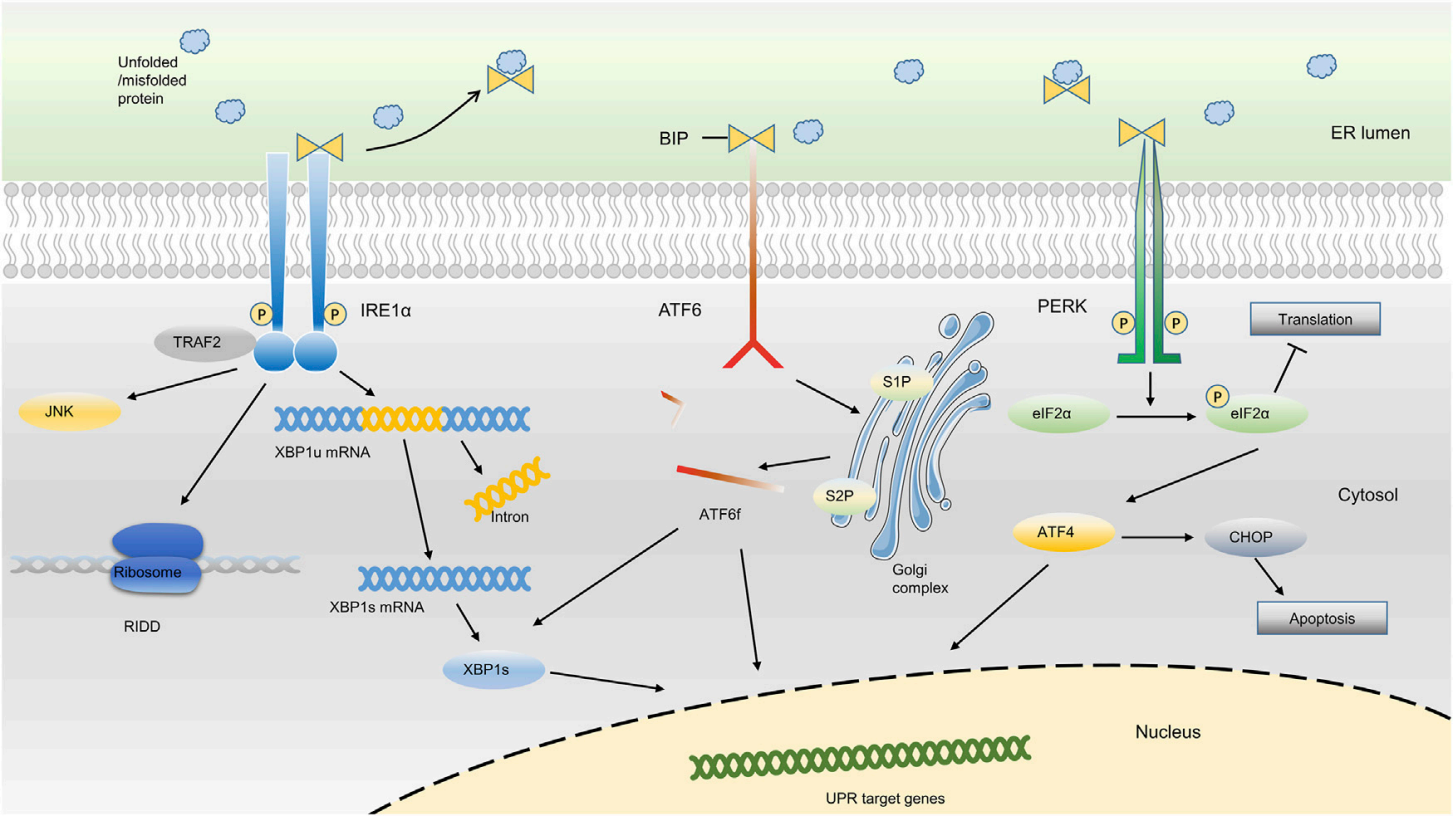
ER stress-sensing and three arms of UPR. Upon ER stress, BiP binds to unfolded proteins in the ER lumen and activates three ER sensors, ultimately resulting in the occurrence of the UPR. The three arms of UPR (PERK, IRE1α and ATF6) activate a series of downstream transcription factors in the cytosol and nucleus, which thus reduce the protein synthesis or promote protein degradation, or ultimately result in cell apoptosis.

To respond to ER stress, cells activate an adaptive response mainly with three distinct arms to reduce the quantity of mismodified proteins, which is called unfolded protein response (UPR) (Hetz, 2012; Senft and Ronai, 2015; Shpilka and Haynes, 2018; Hofmann et al., 2019). As an important responsive mechanism in cells under ER stress, UPR has been implicated in many chronic diseases such as cardiovascular diseases, musculoskeletal disorders, neurodegeneration, and endocrine disease (Ghosh et al., 2019; Hetz et al., 2019; Naiel et al., 2019; Pedersen and Maksymowych, 2019; Yang Y. et al., 2020; Hazari et al., 2020; Ji et al., 2020). Usually, the UPR senses the protein-folding status within the ER lumen and transduces this information to the cytosol and nucleus to relieve overloading of unfolded proteins, which is called adaptive UPR. However, under sustained ER stress conditions, the UPR triggers proapoptotic programs to eliminate existing cells and thus may aggravate the disease course, which is termed as proapoptotic UPR (Senft and Ronai, 2015; Shpilka and Haynes, 2018). The UPR signaling pathway involves two main components: ER stress sensors at the ER membrane and downstream transcription factors in the cytosol and nucleus. The whole picture of ER stress sensors and the downstream transcription factors were detailedly described in Figure 1.

In the absence of ER stress, the ER chaperone binding immunoglobulin protein [BiP, also known as 78-kDa glucose-regulated protein (GRP78) or heat shock protein A5 (HSPA5)], binds to the luminal domain of three ER transmembrane protein sensors, namely inositol-requiring kinase 1α (IRE1α), protein kinase RNA-like ER kinase (PERK), and activating transcription factor 6 (ATF6), and inhibits their activation. Upon ER stress, BiP binds to excessively accumulated misfolded or unfolded proteins in the ER and loses the ability to inhibit the three ER sensors, ultimately resulting in the occurrence of the UPR (Gardner et al., 2013; Senft and Ronai, 2015; Shpilka and Haynes, 2018; Koopman et al., 2019; Kopp et al., 2019; Preissler and Ron, 2019; Siwecka et al., 2019). Based on the different sensors, the UPR is classically divided into three branches: IRE1α, PERK, and ATF6 (Senft and Ronai, 2015; Hetz and Saxena, 2017; Hofmann et al., 2019; Koopman et al., 2019; Mogilenko et al., 2019).

IRE1α, which is evolutionarily conserved in eukaryon, contains RNase and kinase functions under ER stress. Upon activation, IRE1α dimerizes and autotransphosphorylates, acting as a UPR transducer that leads to excision of a 26-nucleotide intron from the mRNA encoding the transcription factor X box-binding protein 1 (XBP1) within the cytosol. After excision, the reading frame of *XBP1* mRNA transforms into an active and stable form, which is termed spliced XBP1 (XBP1s). XBP1s translocates to the nucleus to upregulate its target genes, which support cell survival, restore proteostasis, and activate ER-associated degradation (ERAD) (Calfon et al., 2002; Walter and Ron, 2011; Senft and Ronai, 2015; Chalmers et al., 2017; Hetz and Saxena, 2017). During the ERAD, the misfolded/unfolded proteins are transferred out of ER lumen and then degraded *via* proteasome-dependent pathway. In addition, IRE1α targets a group of endogenous mRNAs for degradation of ER-located proteins and certain microRNAs due to its RNase activity, a process termed regulated IRE1α-dependent decay (RIDD). In RIDD process, decreased mRNA abundance may result in a decreased level of protein folding and thus recover the ER homeostasis (Maurel et al., 2014; Carew et al., 2018; Huang et al., 2019).

In addition, ER stress also triggers the activation of PERK through the dimerization, oligomerization and autophosphorylation of PERK, which inhibits protein synthesis *via* direct phosphorylation of eukaryotic translation initiator factor 2α (eIF2α) (Back et al., 2009; Feng et al., 2014; Abdel-Nour et al., 2019; Balsa et al., 2019). Phosphorylation of eIF2α by PERK subsequently leads to the transcription of the mRNA encoding activating transcription factor 4 (ATF4), which promotes ER adaptation and cell survival (B'Chir et al., 2013; Balsa et al., 2019). PERK signaling also regulates the expression of several microRNAs that attenuate protein translational load or expand ER capacity. For example, miR-30c-2-3p is induced by the PERK pathway of the UPR and governs the expression of XBP1, which promotes secretory capacity and cell survival in the adaptive UPR (Byrd et al., 2012). In addition, PERK-dependent miR-211 induction directly targets the *Chop* promoter to repress C/EBP homologous protein (CHOP) expression and thereby protects cells from apoptotic commitment (Chitnis et al., 2012).

Moreover, ER stress also leads to the activation of ATF6, a member of the transcription factor family containing a basic Leu zipper (bZIP) structure. The ATF6 has two main isoforms, ATF6α and ATF6β (Glembotski et al., 2019). When cells undergo ER stress, ATF6 is readily trafficked from the ER to the Golgi apparatus. After being processed by site 1 protease (S1P) and site 2 protease (S2P), ATF6 is transformed into a cytosolic fragment termed ATF6f and transcriptionally upregulates genes encoding components involved in ERAD and XBP1 within the nucleus (Fass, 2019; Glembotski et al., 2019; Oka et al., 2019; Waldherr et al., 2019). Although the activation of ATF6 and XBP1 belong to different arms of UPR, they can both promote the transcription of genes encoding ER chaperones, and enzymes that help the protein secretion or the degradation of misfolded protein, indicating a potential synergistic effect of different arms of UPR (Shoulders et al., 2013).

Although all three arms of the UPR are crucial for cells to respond to ER stress, these sensors may be differentially activated in different conditions. For example, IRE1α and PERK are rapidly activated when the ER calcium content is depleted in CHO cells (DuRose et al., 2006), whereas reduced glycosylation or altered redox metabolism in HeLa-TetOff cell line preferentially activate ATF6 arm of UPR (Maiuolo et al., 2011). Consequently, it is necessary to distinguish different inducers of ER stress in different disease models.

## ER Stress and the UPR in IDD

Previous studies have reported that ER stress is a major underlying mechanism involved in various musculoskeletal system disorders, such as rheumatoid arthritis (RA) (Yoo et al., 2012; Savic et al., 2014; Rahmati et al., 2018), osteoarthritis (OA) (Hosseinzadeh et al., 2016; Tang et al., 2017), myodegenerative disorders (Kustermann et al., 2018), abnormal bone mass (Collison, 2018; Li et al., 2018), and certain developmental diseases (dwarfishness, spondylolisthesis, etc.) (Woodman, 2013; Mullan et al., 2017; Zheng et al., 2019). Due to abundant synthesis and the secretion of matrix proteins, the ER in IVD cells is highly vulnerable to external stimuli. Moreover, the microenvironment of IVDs is quite complicated, suffering from high osmotic pressure, low pH, oxidative stress and mechanical load (Vergroesen et al., 2015). The increased expression of ER stress-related proteins and ER stress-related apoptosis markers, such as GRP78, CHOP, and caspase 12, have been reported in NP tissues of IDD patients (Zhao et al., 2010; Liao et al., 2019). The comparison of human NP samples with different degrees of degeneration revealed that increased expression levels of GRP78 and CHOP were positively correlated with the Pfirrmann grades of IDD (Liao et al., 2019). These research results strongly indicate that ER stress participates in the process of IDD.

Oxidative stress and inflammatory reaction are two major factors involved in IDD pathogenesis. Accumulation of ROS and proinflammatory factors can induce the degradation of ECM and reduce the number of IVD cells, which leads to the occurrence and development of IDD (Chen et al., 2016; Cheng et al., 2018). H_2_O_2_, a classic oxidative stress inducer, upregulates GRP78, CHOP, and caspase 12 *in vitro* and switches NP cells towards apoptosis, indicating oxidative stress is an ER stress inducer in IDD process (Luo et al., 2019a). Although it is still unclear how ROS signaling induces ER stress in IVDs, some possible mechanisms should be taken into consideration. On one hand, ROS disturbs the correct disulfide bond formation and proper protein folding, which may initiate the ER stress (Wang et al., 2016; Ochoa et al., 2018). On the other hand, ER-mitochondrial Ca^2+^ crosstalk may also play an important role in ROS-induced ER stress (Ochoa et al., 2018; Lin et al., 2021). Moreover, both IL-1 and TNF-α, two well-established proinflammatory cytokines involved in the development of IDD, can also induce ER stress and bias towards apoptotic signaling of IVD cells. Xiaotao Wang et al. verified that 10 ng/ml TNF-α for 24 h triggered the increased expression of BiP and the apoptosis of rat NP cells (Chen et al., 2018; Chen et al., 2019b). Xiangyang Wang et al. reported that 24 h treatment with 75 ng/ml IL-1β caused an increase in two UPR markers, CHOP and ATF6, in passage 2 human NP cells (Xu et al., 2017). Contrary to these findings, Olga *et al.* reported that TNF-α (5 and 10 ng/ml) did not activate ER stress, whereas IL-1β (5 and 10 ng/ml) activated gene and protein expression of GRP78 but did not influence [Ca^2+^]_i_ flux and expression of CHOP in human primary NP cells (Krupkova et al., 2018). These discrepancies may be due to the distinct resources of NP cells or different dosages of IL-1β used.

Although ER stress have been verified an important mediator between inflammation/ROS and IDD, the ER stress can also induce inflammatory condition or ROS accumulation, which may in turn exacerbate the process of IDD induced by these factors. For examples, ER stress can initiate the ligand-independent activation of TRAIL receptors, which then results in caspase-8/FADD/RIPK1-depezndent nuclear factor-kappa B (NF-κB) activation and inflammatory cytokine production (Sullivan et al., 2020). NOD1 and NOD2, two members of the NOD-like receptor family, can also mediate ER stress-induced inflammation (Keestra-Gounder et al., 2016). Lu Chen et al. reported that the activation of XBP1 pathway favored the phosphorylation and nuclear translocation of p65 subunit of NF-κB in NP cells, indicating a potential crosstalk between ER stress and inflammation in the process of IDD (Chen et al., 2019c). In addition, ER stress can also promote ROS production. CHOP was reported to transcriptionally activate Ero1α and thus increase ROS level during ER stress. Moreover, Ero1α causes inositol-1,4,5-trisphosphate receptor (IP3R)-mediated Ca^2+^ leakage from the ER (Anelli et al., 2012). An excess of Ca^2+^ released from the ER can be taken up by mitochondria, which may result in an excessive release of cytochrome C from the mitochondrial matrix and thus enhance the production of ROS (Boehning et al., 2003; Wozniak et al., 2006). ER stress induced by impaired calcium homeostasis was also observed in NP cells, indicating a potential crosstalk between oxidative stress and ER stress in the process of IDD (Luo et al., 2019b).

Excessive mechanical loading is positively correlated with the process of IDD (Adams et al., 2000; Von Forell et al., 2015; Desmoulin et al., 2019). It has been reported that 1.0 MPa static compression was sufficiently high to induce an increase in CHOP, caspase12, and cleaved caspase12, resulting in apoptosis in rat NP cells (Wang et al., 2018). Li et al. reported that 20% surface elongation at a frequency of 6 cycles/min was also an external factor triggering ER stress and promoting NP cells apoptosis (Li et al., 2017). Moreover, supraphysiological tension also increased the expression of CHOP and triggered apoptosis in AF cells (Zhang et al., 2011). These studies indicated that mechanical stress was an ER stress inducer for IVD cells and excessive loading-induced ER stress resulted in the process of IDD. At molecule level, mechanical signaling transduction may mediate the excessive loading-induced ER stress. Piezo type mechanosensitive ion channel component 1 (Piezo1) protein, an important mechanosensitive ion channel, has been reported to participate in the mechanical signal transduction of eukaryotic cells (Zhao et al., 2018). Cao Yang et al*.* reported that increased ECM stiffness promoted the expression of mechanosensitive ion channel PIEZO1 and ER stress markers (GRP78 and CHOP). *Piezo1* knockdown attenuated stiff ECM-induced ER stress and thus inhibited NP cell senescence and apoptosis (Wang et al., 2021). Furthermore, some other mechanosensitive molecules, such as transient receptor potential vallinoid-4 (TRPV4), N-cadherin adhesions, connexin 43 connexons or integrins, were reported to participate in the mechanical signal transduction of IVD cells and disfunction of which was connected with the process of IDD. Whether the mechanosensitive molecules mentioned above involves in mechanical loading-induced ER stress remains to be seen in further experiments.

Metabolic disturbance is another common incentive for the process of IDD. Due to hypoxia, anaerobic glycolysis is the main energy source of NP tissue, which results in an increased lactate production and a decreased pH level. High lactic acid concentration reduces the synthesis rates of matrix protein and promotes the apoptosis of NP cells, which leads to the process of IDD (Wu et al., 2014). Acidic environment was reported to result in an increase in GRP78, CHOP, caspase12, while the blockade of acid-sensing ion channel 1a (ASIC1a) partially alleviated IDD by the inhabitation of ER stress (Xie et al., 2017; Xie et al., 2018). These results indicate acid-sensing ion channel is enrolled in low pH-induced ER stress in IVDs. Moreover, abnormal glucose level is also involved in the occurrence of IDD (Zhang et al., 2019a). In hyperglycemic condition, advanced glycation end products (AGEs) accumulate in peripheral tissue and thus reduce collagen affinity for some key molecules (Gautieri et al., 2014). Addition of AGEs has been proved to increase the expression of ER stress as well as UPR markers and thus induce the apoptosis of NP cells. In addition, glucose deprivation time-dependently upregulated the levels of p-eIF2α and ATF4 in NP cells, suggesting that ER stress is triggered by nutrient deprivation *via* the eIF2α/ATF4 pathway (Chang et al., 2017).

## Potential Therapeutic Strategies for IDD by Targeting ER Stress and UPR

Accumulated evidence suggests that IVDs are easily subject to ER stress, which may play a pivotal role in the process of IDD. Given the role of ER stress and UPR in IVD function and dysfunction, targeting molecules in inhibiting ER stress and modulating UPR is undoubtedly an attractive therapeutic method for disc degeneration.

Given the effect of stabilizing the folded protein and promoting the protein transfer out of the ER, chemical chaperones for reducing the level of unfolded protein in the ER lumen are effective drugs for inhibiting ER stress (Welch and Brown, 1996; Xie et al., 2002). Zengwu Shao et al. reported that tauroursodeoxycholic acid (TUDCA), a FDA-approved bile acid with chaperone properties, was able to alleviate the 1.0 MPa compression-induced apoptosis and necroptosis of NP cells by inhibiting ER stress (Wang et al., 2018). Another FDA-approved chemical chaperone, 4-phenylbutyricacid (4-PBA), has also been verified to alleviate IDD by inhibiting ER stress in different experiment condition, such as the application of cyclic tension on AF cells and the treatment of AGEs, H_2_O_2_, or TNF-α on NP cells (Chen et al., 2018; Chen J. et al., 2019; Luo et al., 2019a; Luo et al., 2019b). These results indicated that inhibiting ER stress by these chemical chaperones is a promising strategy for ameliorate the process of IDD.

Targeting molecules in the three arms of UPR to inhibit IVD cell death induced by excessive ER stress might be promising for future IDD therapy. Takeshi Fujii et al. reported that the PERK-ATF4 pathway was activated in human degenerative IVD tissues (Fujii et al., 2018). Pharmacological inhibition of PERK (GSK2606414) significantly suppressed the starvation-induced expression of TNF, ADAMTS5 transcripts and apoptosis in human AF cells. This result indicated that PERK inhibitors might be promising drugs for IDD (Fujii et al., 2018). However, Lu Chen et al. reported that both PERK inhibitor and IRE1 inhibitor increased cell apoptosis induced by TNF-α and reduced cell proliferation of NP cells, which seemed contradicted to the previous result (Chen et al., 2018). On the one hand, the discrepancy between the two results could attribute to the different cell types as well as *in vitro* models. On the other hand, PERK inhibitor used in the two studies was reported to inhibit cellular RIPK1 at a lower concentration, indicating some nonspecific effects of these inhibitors (Rojas-Rivera et al., 2017). In addition, previous studies showed that silencing some molecules in the downstream of UPR, such as ATF4 and CHOP, successfully reduced the expression of IL-6 and prevented the process of IDD, indicating that manipulating some molecules in UPR pathway could be a potential molecular target for IDD prevention (Fujii et al., 2018; Krupkova et al., 2018). Recently, Dike Ruan et al. reported that knockdown of IRE1-α or PERK was able to recover the dampened ECM synthesis induced by TNF- and IL-1, while knockdown of ATF6 showed no protective effect in this inflammation-induced model (Wen et al., 2021). However, ATF6 small interfering RNA (siRNA) markedly inhibited the tert-butyl hydroperoxide-induced apoptosis of CEP cells, indicating its protective effect in an oxidative stress-induced model (Xie et al., 2020). These results implied that not all three arms of UPR were simultaneously involved in the IDD induced by single pathogenic factor. It is very important to determine which sub-pathway is involved in a certain pathogenic process and provide personalized treatment.

Targeting autophagy-associated molecules might be another effective method for alleviating the process of IDD induced by overwhelming ER stress. Once the UPR pathway cannot alleviate the ER stress, autophagy and even apoptosis are eventually activated (Hetz, 2012). The PERK-eIF2α-ATF4 pathway has been confirmed to help the formation of the autophagosome. ATF4 and CHOP transcriptionally regulate numerous autophagy-associated genes (ATGs) by binding these factors to specific promoter *cis* elements of ATGs (B'Chir et al., 2013). In addition, the IRE1 arm also plays an important role in the activation of autophagy. Activation of IRE1α recruits the adaptor protein TNF receptor-associated factor 2 (TRAF2), which activates the apoptosis signal-regulating kinase 1 (ASK1) pathway and its downstream target JUN N-terminal kinase (JNK), enabling the dissociation of Beclin-1 (an essential autophagy regulator), activation of PI3K signaling, and triggering autophagy (Deegan et al., 2013; Liu et al., 2020). Meiqing Wang et al. showed that activating autophagy by rapamycin, an inhibitor of MTORC1, markedly reduced p-EIF2AK3-mediated ER stress-apoptosis in flow fluid shear stress-treated chondrocytes. This result implied that autophagy activation might be a promising strategy for inhibiting ER stress in IDD process (Yang H. et al., 2020). It has been reported that 1.0 MPa static compression is sufficiently high to trigger ER stress and promote the conversion of LC3B-I to LC3B-II (hallmark at an early stage of autophagy) (Ma et al., 2013; Wang et al., 2018). Transmission electron microscope (TEM) images also revealed that both the expansive endoplasmic reticula and autophagosomes were present in NP cells following exposure to 1.0 MPa for 36 h (Ma et al., 2013). In addition, knockdown of ATF4 with siRNA significantly attenuated the conversion of LC3-I to LC3-II, which also confirmed the link between the UPR and autophagy in IDD (Chang et al., 2017). Up to now, many small-molecule modulators of mammalian autophagy have been verified to alleviated IDD, indicating a huge potential for medical application (Chen et al., 2016; Zhang et al., 2019b; Wang et al., 2020). Exosomes derived from bone marrow mesenchymal stem cells also showed potential therapeutic effect for IDD model by activating autophagy and inhibiting ER stress (Liao et al., 2019; Shi et al., 2021), Some promising therapeutic treatment targeting ER stress pathway has been listed in Table 1.

**TABLE 1:** Promising therapeutic treatment targeting ER stress.

Categories	Name	Mechanism of action	Ref
Chemical chaperones	TUDCA	Reducing unfolded protein in ER	Wang et al. (2018)
	4-PBA		(Chen et al., 2018; Chen et al., 2019a; Luo et al., 2019a; Luo et al., 2019b)
UPR regulators	GSK2606414	PERK inhibitor	Fujii et al. (2018)
	siRNA for PERK	Inhibiting PERK-eIF2α-ATF4-CHOP pathway	Wen et al. (2021)
	siRNA for ATF4		Fujii et al. (2018)
	siRNA for CHOP		(Fujii et al., 2018; Krupkova et al., 2018)
	siRNA for IRE1-α	Inhibiting IRE1-α arm	Wen et al. (2021)
	siRNA for ATF6	Inhibiting ATF6 arm	Xie et al. (2020)
Autophagy regulator	Exosomes	Activating autophagy and inhibiting ER stress	(Liao et al., 2019; Shi et al., 2021)
	Rapamycin	Inducing p-EIF2AK3-mediated ER stress	Yang et al. (2020a)

## Discussion and Prospects

IDD is a major cause of LBP that presents an important scientific and social issue and has received substantial attention. DALYs and YLDs caused by this disc degenerative disease have imposed huge burden for the individuals and the whole society (Diseases and Injuries, 2020; Cieza et al., 2021). Due to the lack of efficient interventions to prevent the occurrence or halt the progression of IDD, it is necessary for us to figure out molecule mechanism of IDD and find out some effective therapeutic treatment.

**FIGURE 2:**
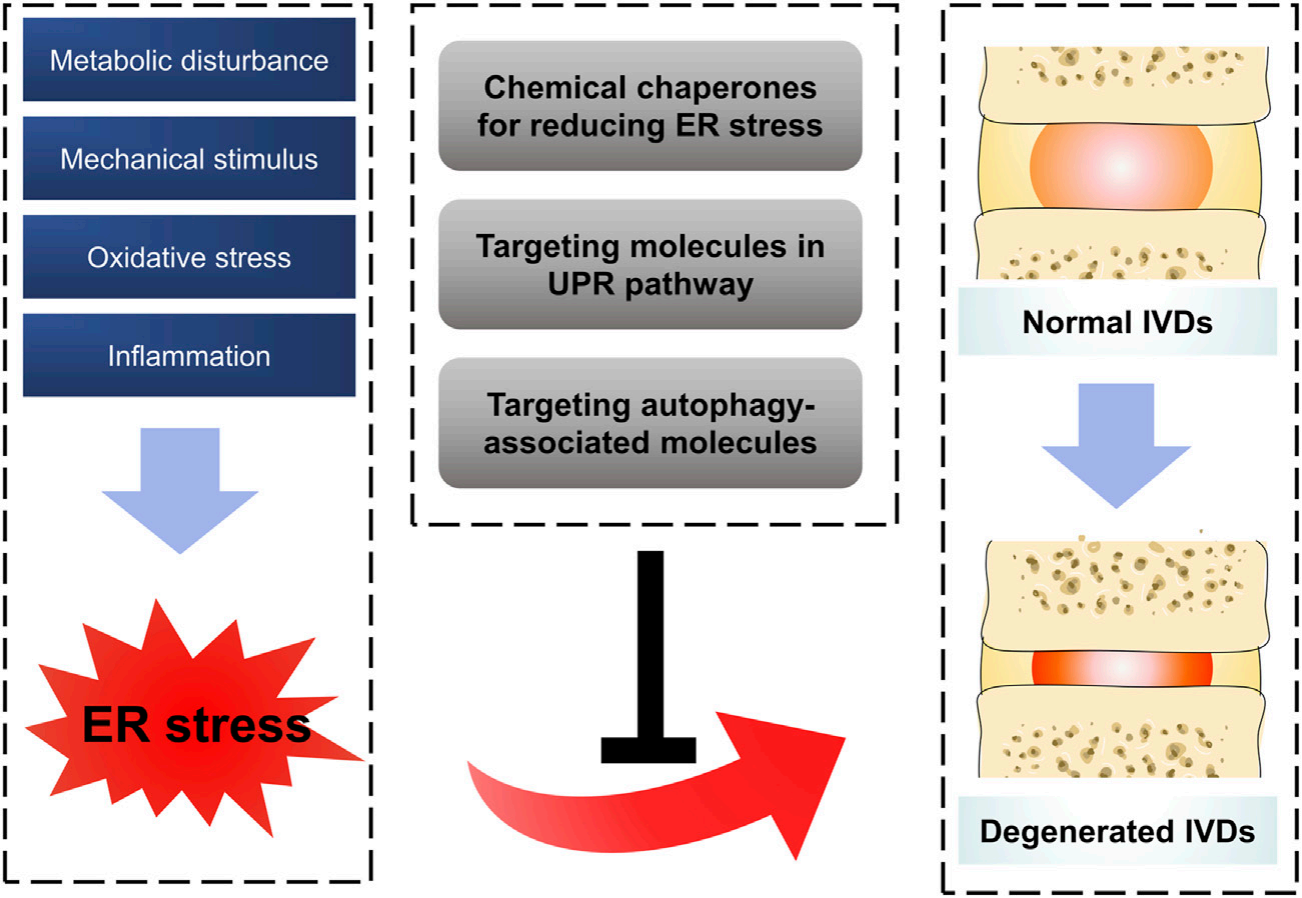
Schematic diagram for targeting ER stress to prevent IDD. Oxidative stress, inflammation, mechanical stimulus and metabolic disturbance are common factors affecting IVDs, which were confirmed to trigger ER stress in IDD process by a series of reasonable evidence. Some therapeutic strategies by targeting ER stress showed promising effect on ameliorating the process of IDD.

The risk factors of IDD are complicated and its pathogenesis is still unclear. As large polymerized molecules in the ECM, collagens undergo extensive post-translational modifications and folding in the ER, which pose susceptibility to ER stress. Over the past 10 years, studies have indicated that ER stress results in aberrant IVD cell functions, such as decreased cell proliferation and ECM synthesis, increased cell apoptosis, senescence, ECM degradation, and secretion of various inflammatory cytokines. In order to handle the impact of ER stress, the activation of the UPR and other defensive processes orchestrates diverse pathways to recover IVD cell functions (Senft and Ronai, 2015; Krupkova et al., 2018; Chen et al., 2019b; Liao et al., 2019; Luo et al., 2019a; Luo et al., 2019b). This review summarized the relationship between ER stress and IDD and highlighted potential therapeutic targets for treating this condition (Figure 2). Although there is still a long way to realize the clinical transformation for these therapeutic strategies, drugs targeting ER stress pathway showed their charming potential. On the one hand, more studies in recent years attempted to illustrate the exact mechanism between ER stress and the pathogenesis of IDD, and more effective therapeutic strategies have been verified in pre-clinical experiments (Chen et al., 2019c; Liao et al., 2019). On the other hand, some drugs targeting ER stress have been accepted in clinical trials in patients and show some promising outcomes. For example, Bip administration showed the effectiveness and safety in patients with active RA. Compared with placebo group, patients in 5 or 15 mg Bip group showed significantly lower serum concentrations of C-reactive protein after 2-week treatment (Kirkham et al., 2016). Numerous interventional measures targeting ER stress pathway have been adopted in some pre-clinical experiments and even clinical trials, however, there still some tough challenges for the process of clinical transformation. First of all, among the limitations of these clinical trials were their small sample size as they were designed as randomized and controlled experiment, indicating a dampening credibility for their results. Furthermore, some local delivery systems with noninvasive or less invasive characteristics remain to be explored for the purpose of improving drugs’ efficiency and reducing their side effects. Last but not least, there are still some questions to be solved in ER stress response mechanism. Whether can the aberrant ECM components (collagens or proteoglycans) directly trigger the ER stress in IDD process? Do all three arms of UPR play a role in ER stress-induced IDD in a coordinated manner? What is the overall perspective of the crosstalk between IDD inducers and response mechanism of ER stress in the development of IDD? For screening out special therapeutic drugs, it is quite helpful to address these problems.

Data suggest that ER stress is a pivotal mediator in the pathogenesis of IDD in many recent experiments, whereas the direct evidence is still limited. For example, most studies paid their attention on the activation of ER stress or UPR pathway in IVDs via the expression of some ER stress or UPR markers, such as GRP78, CHOP or caspase 12, few studies showed the accumulation of misfolded protein in IVD cells in the process of IDD. To date, most ER stress-mediated IDD experiments *in vitro* have been performed on NP cells. Limited attention has been paid to ER stress-mediated degeneration of the AF, CEP, and even the entire structure of the functional spinal unit. Moreover, the exact relationships among different mechanisms responsible for IDD require further elucidation. For example, in spite of UPR, many ER stress-related models *in vitro* also showed some subcellular changes related to autophagy and mitochondrial problems (Hetz, 2012; Senft and Ronai, 2015). Cao Yang et al. also reported that berberine, an isoquinoline alkaloid usually functioning by modulating autophagy or mitochondrial dynamics, successfully inhibited the process of IDD by inhibiting ER stress. These results hinted that the crosstalk among different mechanisms of cell response might impose a comprehensive effect on maintaining physiological homeostasis for IVD cells. Thus, some small molecule drugs with a broad spectrum of activity these cellular responses, such as melatonin, quercetin, resveratrol or curcumin, may have a wide range of potential applications on IDD prevention. Based on our comprehensive understanding of ER stress and its responsible mechanism in IDD, some translational approaches (the clinical use of targeted therapy, exosomes from stem cells, potential conventional drugs, etc.) are promising.

## Conclusion

The research on the relationship between ER stress and the pathogenesis of IDD is still in its infancy, with a growing number of studies available supporting this view. Our work summarized the current knowledge regarding the impact of ER stress on the process of IDD, as well as some potential therapeutic methods for alleviating IDD by targeting ER stress pathway. Further studies to explore ER stress and its responsible mechanisms in IDD may offer more efficient therapeutic strategies to prevent or halt the process of disc degenerative diseases.
